# Consensus on the prevention and repair of titanium mesh exposed wound after cranioplasty (2024 edition)

**DOI:** 10.1093/burnst/tkae055

**Published:** 2024-10-23

**Authors:** Pihong Zhang, Xiaobing Fu, Yuesheng Huang, Wound Repair Professional Committee of Chinese Medical Doctor Association

**Affiliations:** Department of Burns and Plastic Surgery, Xiangya Hospital of Central South University, No. 87 Xiangya Road, Kaifu District, Changsha 410008, Hunan Province, China; Research Center for Wound Repair and Tissue Regeneration, Medical Innovation Research Department, the PLA General Hospital, No. 28 Fuxing Road, Haidian District, Beijing 100853, China; Institute of Wound Repair and Regeneration Medicine, Southern University of Science and Technology School of Medicine, and Department of Wound Repair, Southern University of Science and Technology Hospital, Shenzhen 518055, China

**Keywords:** Head injuries/penetrating, Postoperative complications, Surgical flaps, Cranioplasty, Titanium mesh, Wound repair, Expert consensus

## Abstract

Titanium mesh exposure after cranioplasty is the most serious complication of this procedure. Although some clinical experience has been gradually accumulated over the years in the diagnosis and treatment of titanium mesh exposure, the treatment is often not standardized and it is difficult to achieve satisfactory repair results due to insufficient understanding of its pathogenesis and concurrent infections. To normalize the diagnosis and treatment of titanium mesh exposed wounds after cranioplasty and improve the therapeutic effect and the quality of life of patients, the Wound Repair Professional Committee of Chinese Medical Doctor Association organized an expert discussion based on the literature and current diagnosis and treatment status of titanium mesh exposed wounds after cranioplasty at home and abroad, and reached a consensus on the pathogenesis, preventive measures, and diagnosis and treatment strategies of titanium mesh exposed wounds after cranioplasty to provide reference for relevant clinicians.

Highlights

- To improve the treatment effect of titanium mesh exposed wounds after cranioplasty and the quality of life of patients, based on relevant literature evidence and clinical practice, combined with the experience and viewpoints of experts in the field of wound repair, a consensus was formed on the prevention and wound repair of titanium mesh exposure after cranioplasty, providing academic basis and guiding opinions for the prevention and treatment of titanium mesh exposed wounds after cranioplasty.
- This is the first expert consensus on the prevention and wound repair of titanium mesh exposure after cranioplasty at home and abroad, which has quite strong academic and practical guidance value.

## Background

Full-thickness cranial bone defects are common sequelae following severe traumatic brain injuries, cerebrovascular accidents, and intracranial or cranial tumor surgeries. This condition is frequently accompanied by a constellation of neurological symptoms known as motor trephine syndrome or sunken brain and scalp flap syndrome. These symptoms include headaches, dizziness, irritability, fatigue, memory loss, depression, etc., which significantly impact the overall quality of life of patients [1]. To restore cranial integrity and prevent reinjury to brain tissue while maintaining stable intracranial pressure, cerebrospinal fluid dynamics, and cerebral hemodynamics, as well as improving brain nerve function and restoring patients’ normal craniofacial appearance, cranioplasty should be performed for defects with an area > 3 cm^2^ [2–4].

Cranioplasty is a routine and simple procedure, with titanium mesh commonly employed as the repair material; however, postoperative complications may occur, particularly titanium mesh exposure. In recent years, the use of customized titanium mesh based on computed tomography (CT) scans has become a widely adopted approach for accurately repairing skull defects [5]. Nevertheless, factors such as a thin scalp, compromised blood supply, infection, and physical stress often contribute to the occurrence of titanium mesh exposure [6, 7]. Failure to promptly or appropriately address this issue may result in wound expansion or complicated infections with potential invasion of deep tissues by bacteria, leading to life-threatening intracranial infections. Consequently, subsequent treatment and repair may become more challenging. The field of neurotraumatology has reached an international consensus and put forth an initial set of practical consensus-based clinical recommendations for posttraumatic cranioplasty, with a specific focus on surgical timing, material selection, complications, and surgical procedures [8].

Given that titanium mesh-exposed wounds after cranioplasty are primarily managed by the burn and plastic surgery or wound repair surgery departments, a great deal of clinical experience has accumulated in terms of infection control, retention of titanium mesh, and wound repair. However, the clinical diagnosis and treatment of titanium mesh-exposed wounds following cranioplasty still encounter many challenges, with a dearth of evidence-based medical guidelines for wound assessment and management. Based on the existing literature and the current diagnosis and treatment of titanium mesh exposure after cranioplasty, a consensus was reached regarding the occurrence mechanism, preventive measures, and diagnostic and therapeutic strategies for managing titanium mesh exposure, providing valuable insights for clinical practice.

## Methods

### Consensus working group

The consensus working group consisted of experts specializing in burn and plastic surgery, wound repair, neurosurgery, evidence-based medicine, and other relevant disciplines. Moreover, the consensus working group was divided into the following subgroups: consultant and team leader, expert committee group, writing group, clinical problem solicitation expert group, and evidence evaluation and secretary group. The roles and duties assigned to each group were explicitly defined to facilitate the achievement of this consensus. In addition, the evidence evaluation and secretary group wrote a proposal that defined the composition of the consensus working group, the presentation and collection of clinical issues, the evaluation and summarization of the evidence, the generation of recommended opinions, users and the target population, and the publication and updates of the consensus.

### Systematic literature review and level of evidence determination

A comprehensive literature search was conducted to identify high-quality studies focusing on the etiology of titanium mesh exposure and wound management after cranioplasty. PubMed, Web of Science, Embase, Cochrane Library, CNKI, and Wanfang Data were searched using the keywords ‘cranioplasty’ and ‘titanium mesh’, with the search period limited to 1 April 2024. The included literature included systematic reviews, randomized controlled trials, observational studies (cohort studies, case control studies, cross-sectional studies, case series reports, etc.), and expert opinion. After carefully reading the title and abstract for preliminary screening, a meticulous evaluation of the full texts of the relevant studies was conducted, followed by a quality assessment of the included literature using the critical appraisal skills programme. The evidence was classified according to the Oxford Centre for Evidence-Based Medicine 2011 Levels of Evidence (Table 1) [9–11].

**Table 1:** The 2011 Oxford Centre for Evidence-Based Medicine Levels of evidence

**Level of evidence**	**Description**
**I**	Systematic review of randomized trials or *n*-of-1 trials
**II**	Randomized trial or observational study with dramatic effect
**III**	Non-randomized controlled cohort/follow-up study
**IV**	Case-series, case-control studies, or historically controlled studies
**V**	Mechanism-based reasoning

### Formation of recommendation and determination of recommendation strength

The level of agreement was assessed using a three-point Likert scale (agree, uncertain, and disagree). According to the Delphi expert investigation method, each expert was required to select a single option based on their level of agreement for each recommendation. Following the initial feedback from the expert committee group, the evidence evaluation and secretary group compiled statistics and summaries to assign appropriate recommendation levels. Several detailed recommendations from the first round of Delphi expert investigation were revised and integrated through discussions and individual communication within the expert group. Ultimately, the evidence evaluation and secretary group incorporated and modified certain content on the basis of feedback from the expert committee group before submitting it for a second round of Delphi expert investigation.

A total of 27 experts, representing diverse disciplines including burn surgery, wound repair, plastic surgery, and neurosurgery, participated in two rounds of Delphi expert investigation and two rounds of consensus seminars. Following extensive discussions and deliberations, a consensus was ultimately reached by Chinese experts on the prevention and repair of titanium mesh-exposed wounds after cranioplasty. The recommendations that garnered support from >90% of the experts were categorized as ‘highly recommended’, those with agreements ranging from 70 to 90% were labeled ‘recommended’, and those with <70% agreement were excluded from the consensus.

## Clinical issues and recommendations

### Clinical issue 1

Risk of titanium mesh exposure after cranioplasty and its influencing factors.

### Recommendation 1

Considering the potential risk of titanium mesh exposure after cranioplasty, cautious utilization of titanium mesh is recommended for high-risk patients with soft tissue defects. Other influencing factors include large bone defects, severe scalp depression, delayed repairs, thin scalp coverage, excessive suture tension, suboptimal incision alignment, and localized exudation or infection. (Grade of recommendation: recommended; level of evidence: III.)

#### Rationale

Over the past two decades, titanium mesh has been extensively used by neurosurgeons and plastic surgeons for cranial defect repair. Prior to surgery, 3D digital technology and rapid prototyping techniques facilitated the precise prefabrication of titanium mesh implants that accurately conform to the shape of skull defects. This approach is characterized by its straightforward operative procedure, effortless implant fixation during surgery, and satisfactory postoperative cranial contour [5]. However, the placement of the titanium mesh may result in prolonged abrasion of the surrounding soft tissue, leading to scalp thinning and subsequent exposure of the titanium mesh. Mukherjee *et al.* [12] reported that among 174 patients who underwent cranioplasty utilizing titanium mesh, reoperation was necessary for removal of the titanium mesh in 18 patients (10.3%). Maqbool *et al.* [13] reported an incidence rate of exposed implants of 14% (7 out of 50 cases) in patients with skull defects repaired using titanium mesh. Moreover, preoperative radiotherapy and soft tissue atrophy were identified as factors that increase the possibility of titanium mesh exposure. Yeap *et al.* [14] observed a prevalence rate of 17% (15 out of 88 cases) for titanium mesh exposure, while Williams *et al.* [15] reported that complete failure requiring implant removal was observed in only 4% (6 out of 151) of patients. Cabraja *et al.* [16] reported a retrospective study involving 26 patients who underwent cranioplasty using computer-assisted design/computer-assisted modeled titanium implants with an average diameter of 112 mm and were followed up for a period ranging from 6 to 12 years, during which no instances of implant removal occurred.

When comparing the efficacy of polyetheretherketone (PEEK) material for repairing skull defects, multicenter retrospective studies demonstrated a significantly lower incidence of implant exposure in the PEEK group than in the titanium group, along with greater patient satisfaction [17, 18]. Thien *et al*.’s [19] study reported a greater exposure rate of titanium mesh (13.9%) than PEEK among 108 patients; however, it is important to note that PEEK is associated with higher costs [17, 20]. However, more multicenter clinical studies are needed for a comprehensive comparative analysis of cranioplasty materials. Kwiecien *et al.* [21], in their long-term follow-up study involving 130 patients who underwent cranioplasty with titanium mesh, observed only 6 patients (4.6%) with exposed titanium mesh; nevertheless, caution should be exercised when using titanium mesh for high-risk patients with soft-tissue defects during cranioplasty.

The exposure of titanium mesh commonly occurs >6 months after cranioplasty, predominantly affecting the temporal, frontal, and superior regions. These areas are typically characterized by excessive suture tension during incision closure, suboptimal incision alignment, thin soft-tissue coverage, and susceptibility to compression or collision [8]. Zanaty *et al.* [22] conducted a retrospective analysis of clinical data from 348 patients who underwent cranioplasty; the overall complication rate was 31.32% (109 of 348). The study revealed that risk factors for periprocedural complications following cranioplasty include diabetes mellitus, bifrontal cranioplasty, and repeated surgery for hematoma evacuation. It is widely acknowledged that an increased possibility of titanium mesh exposure is associated with advanced age, larger skull defects, longer craniectomy–cranioplasty intervals, more severe bone window depression, thinner scalp tissue, excessive suture tension, scar formation, local tissue dystrophy and thin intraoperative flap separation, or injury to vital blood vessels [8, 23, 24].

### Clinical issue 2

Role of intracranial hypotension in the formation of titanium mesh exposure after cranioplasty.

### Recommendation 2

Considering the strong association between intracranial hypotension and titanium mesh exposure after cranioplasty, it is crucial to proactively prevent postoperative occurrences of intracranial hypotension. (Grade of recommendation: recommended; level of evidence: V.)

#### Rationale

After decompressive craniotomy, cerebral tissue edema subsides, accompanied by softening and atrophy of the cerebral tissue, decreased cerebrospinal fluid secretion, excessive hydrocephalus shunting, etc., which can result in a reduction in cranial contents and intracranial hypotension. Particularly in patients undergoing delayed cranioplasty, the depression of the scalp within the bone window is severe prior to surgery, while the cranial cavity significantly expands after delayed cranioplasty, further reducing intracranial pressure [25]. Additionally, a sustained decrease in intracranial pressure may occur when patients remain standing for an extended period after surgery. The reduced intracranial pressure generates internal suction on the skin flaps at the site of skull repair. On the one hand, 3D titanium mesh frames are characterized by large and relatively sharp mesh holes that lead to chronic abrasion on the scalp. The skin flaps gradually migrate inward through these mesh holes, while the soft tissue covering the titanium mesh undergoes progressive thinning. Consequently, a gradual shallowing of the 3D titanium mesh occurs, accompanied by localized changes resembling an orange peel texture or faint visibility of subcutaneous titanium mesh. On the other hand, internal suction exerts tension on arteriovenous vessels within the flaps, resulting in ischemia, atrophy, and thinning of the flaps, ultimately leading to exposure of the titanium mesh.

Kwiecien *et al.* [21] reported that a substantial proportion (44%) of patients who underwent cranioplasty using titanium mesh experienced scalp atrophy, which was most pronounced within the first 2 years after surgery. This resulted in an ~40% reduction in thickness compared to the preoperative levels, which continued to deteriorate over time. CT scans revealed the presence of dead space in the epidural region among patients with exposed titanium mesh [13]. Further investigations demonstrated that a pressure gradient between the atmosphere and intracranial space could generate dynamic stress on tissues within and adjacent to the mesh, leading to damage and thinning of the scalp tissue, ultimately resulting in implant exposure [26]. Scholars both domestically and internationally have documented the proliferation of epithelial tissue beneath the titanium mesh within the area of skin defects in patients with titanium mesh exposure. Subsequent pathological examination confirmed the presence of dermal appendages, attributed to the growth and integration of skin islands adhering to the meninges [27, 28].

Researchers utilized CT angiography to compare changes in the blood supply of the head flaps before and after cranioplasty. Early cranioplasty did not significantly impact the scalp flap hemodynamics [29]. However, perfusion images of patients with titanium mesh exposure after cranioplasty exhibited a notable decrease in blood supply on the necrotic side of the scalp compared to that on the healthy side. In cases of severe scalp depression and low cranial pressure, the use of chimeric muscle flaps beneath the titanium mesh may effectively prevent local infection and flap thinning caused by internal suction [30, 31]. Some scholars have reported that autologous fat transplantation can effectively improve scalp atrophy and thinning, thereby preventing the exposure of titanium mesh [32]. For patients with a large bone defect and substantial skin flap depression prior to surgery, elevation of the bone window flap can be achieved by adopting a head-down position during and after surgery. This approach effectively prevents low intracranial pressure, allowing for better adherence between soft tissue on the brain surface and the inner aspect of the titanium mesh. As a result, it reduces exudate accumulation under the titanium mesh and facilitates tissue healing [33].

### Clinical issue 3

Relationship between the curvature stress and exposure of titanium mesh after cranioplasty.

### Recommendation 3

For patients with severe local scalp depression and anticipated high skin tension after titanium mesh implantation, it is recommended to perform scalp expansion prior to cranioplasty using titanium mesh in order to accommodate the curvature stress exerted by the implant and prevent postoperative titanium mesh exposure. (Grade of recommendation: highly recommended; level of evidence: III.)

#### Rationale

Although customized 3D titanium mesh exerts significantly lower curvature stress than does ordinary titanium mesh [34], it is crucial to acknowledge the presence of residual curvature stress, which also has the potential to induce continuous pressure on flaps. Such pressure could lead to arterial and venous stretching and thinning within flaps, potentially compromising the blood supply to the flaps. As a consequence, there is a risk for necrosis development and subsequent exposure of the underlying titanium mesh. Notably, increasing the skull defect surface area corresponds with heightened curvature stress from titanium mesh onto flaps. This curvature stress can impact the local blood circulation of the flap similarly to the aforementioned internal suction. Customizing the titanium mesh allows for a reduction in its curvature, facilitating tension-free suturing of the skin flaps. If necessary, an acellular dermal matrix can be employed to augment the tensile strength of the scalp [35]. In cases where patients present with severe depression of the scalp flaps before cranioplasty and an anticipated increase in skin tension after cranioplasty, it is recommended to employ scalp expansion prior to cranioplasty to generate adequate scalp tissue to minimize postcranioplasty skin tension and mitigate the risk of exposure of the titanium mesh [36].

### Clinical issue 4

Relationship between local compression or collision and titanium mesh exposure after cranioplasty.

### Recommendation 4

It is recommended to develop the habit of adopting a healthy lateral lying posture after cranioplasty to prevent titanium mesh exposure resulting from local compression or external collisions. (Grade of recommendation: highly recommended; level of evidence: V.)

#### Rationale

Due to the rigid texture of the titanium mesh frame, local compression not only significantly reduces the blood supply to the surface skin but also exposes the surrounding tissue to twisting and friction. The accumulation of such damage can ultimately lead to exposure of the titanium mesh. Therefore, it is crucial to exercise caution in preventing pressure on the surgical area after cranioplasty with titanium mesh, particularly during periods of sleep and rest, by adopting a healthy lateral lying posture. Excessive pressure dressing after cranioplasty may result in scalp ischemia or even necrosis, leading to the exposure of titanium mesh; therefore, it is necessary to apply dressing with appropriate strength. Additionally, external collision on the surgical area can cause contusion, damage, necrosis, or concurrent infection in local scalp tissue while also potentially causing loosening, deformation and warping at the edges of the titanium mesh. This could subsequently result in cutting through the local skin tissue and exposing the underlying titanium mesh structure, thus emphasizing the importance of protection and avoiding external collisions.

### Clinical issue 5

Influence of local exudation on the exposure of the titanium mesh after cranioplasty.

### Recommendation 5

It is recommended to implement effective drainage, regular dressing changes, appropriate pressure management, and other measures to actively prevent and treat the long-term accumulation of local exudation after cranioplasty while being cautious about the exposure of titanium mesh caused by concurrent infection. (Grade of recommendation: highly recommended; level of evidence: V.)

#### Rationale

After cranioplasty with titanium mesh, incomplete restoration of the initially compressed brain tissue at the bone window may occur, and there may still be a depression on the surface of the brain tissue, resulting in a large cavity under the titanium mesh and subsequent formation of local exudation. Moreover, the inadequate biocompatibility of foreign implants can trigger immune rejection, causing inflammatory responses and fluid accumulation. Alternatively, the thermal effect of electrotomes can potentially lead to soft-tissue injury, resulting in tissue liquefaction and subsequent subcutaneous exudation. Postoperative cranial CT imaging enables clear visualization of exudation beneath the titanium mesh, which can gradually be absorbed by surrounding tissue [6, 8]. However, it is crucial to maintain vigilance regarding potential infections, particularly during the early postoperative period. It should be noted that chronic nonabsorption exudation may flow through incisions or weak areas, resulting in exposure of the titanium mesh. If a further severe infection occurs in the surgical area, removal of the titanium mesh may be necessary, leading to cranioplasty failure. Therefore, proactive and efficient management should be implemented for patients with chronic wound exudation. Moreover, patients should be able to accurately comprehend their condition and strive to enhance medical compliance for optimal outcomes.

### Clinical issue 6

Infection control of the titanium mesh-exposed wounds after titanium cranioplasty.

### Recommendation 6

It is recommended to promptly control the infection of titanium mesh-exposed wounds after cranioplasty, which includes local irrigation and drainage, regular dressing changes, and the scientific and standardized use of negative pressure wound therapy when deemed necessary. (Grade of recommendation: highly recommended; level of evidence: V.)

#### Rationale

Once the titanium mesh is exposed, the initial step is to promptly identify the cause and initiate early treatment. This involves comprehending the etiology of skull defects, the treatment protocols, and the repair duration, as well as investigating the occurrence, progression, and history of the titanium mesh exposure. Moreover, it is crucial to expeditiously remove the patient’s hair to facilitate wound visualization and subsequent comprehensive evaluation through CT scans and other examinations for assessing local exudation beneath the titanium mesh, detecting the presence of infection, and determining the location and size of the skull defects, while also evaluating any indications of ventricular dilation, encephalomalacia, or brain atrophy. When signs of infection, such as erythema, edema, warmth, pain, or exudation at the site of the exposed titanium mesh, are observed, it is recommended that local debridement and dressing changes along with irrigation and drainage be performed. The exudates should be collected for bacterial culture and antibiotic sensitivity tests. If necessary, appropriate antibiotics or broad-spectrum antibiotics against gram-positive bacteria should be administered for systemic anti-infection treatment. Subsequently, antibiotic therapy should be adjusted based on the corresponding results to effectively control the infection. Additionally, malnourished individuals must be provided with nutritional support through the promotion of the consumption of nutrient-rich foods (easily digestible substances high in protein, calories and vitamins and containing crude fiber) to enhance physical function and bolster resistance against infections [6, 37]. Blood sugar levels need to be strictly regulated in diabetic patients.

When dressing the wounds, it is essential to remove the dry scab on the surface, eliminate any necrotic tissue and knots, cleanse the wounds and the latent space beneath the titanium mesh with hydrogen peroxide and normal saline daily, and then cover the wounds with wet iodophor gauze. If necessary, the implementation of vacuum sealing drainage (VSD) can be considered; however, ensuring the integrity of the endocranium beneath the titanium mesh before utilizing VSD is of utmost importance. VSD serves as an effective method for timely aspiration of inflammatory exudates from wounds, enabling efficient control of local infection and promoting granulation tissue formation between the titanium mesh and endocranium while preparing for subsequent wound repair surgery, thereby improving the success rate of the operation. Moreover, when combined with debridement and irrigation with chymotrypsin, VSD can effectively control implant-related infection without necessitating the replacement of the implant [38]. It is important to cautiously control the application of negative pressure for treating titanium mesh-exposed wounds, and the absolute value of negative pressure is generally set at ≤125 mmHg [38, 39]. In order to prevent exacerbation of the external pressure on the local flaps, it is not recommended to use VSD for wounds in regions where the local scalp exhibits obvious thinning due to intracranial hypotension-induced internal suction and where the faint outline of the titanium mesh can be observed around the wounds.

### Clinical issue 7

Trade-off associated with the decision to retain the exposed titanium mesh in the wound treatment after cranioplasty.

### Recommendation 7

After fully communicating with patients and their families, the titanium mesh should be retained completely if the exposed area is small, the duration of exposure has been short, and there are no apparent signs of infection. In instances where there is limited infection or tissue necrosis beneath the titanium mesh, appropriate debridement should be performed while partially retaining the titanium mesh. However, if there is extensive pus formation or repeated exposure that cannot be controlled, complete removal of the titanium mesh is recommended. (Grade of recommendation: highly recommended; level of evidence: III.)

#### Rationale

As a cranioplasty material, titanium mesh is thin and has excellent biocompatibility. Mesh holes can promote the growth of granulation tissue and establish reliable vascularization between the flaps and meningeal tissue, enabling the retention of the titanium mesh even after exposure. Some scholars [40] have conducted research on cases where narrow strip necrosis occurs at the edge of the flap incisions due to insufficient blood supply or excessive tension in the early stages following cranioplasty with digitally shaped titanium mesh. Standard dressing treatment can be employed to promote granulation tissue growth to cover the titanium mesh in cases where there is no infection or apparent exposure. The regenerated epidermis grows from the edges of the wounds, achieving healing under scabs. For patients who present with limited areas of necrosis at the margins of the flaps and exposed titanium mesh, tension-free suturing can be performed at the site of the soft-tissue relaxation after thorough debridement. In cases where direct suturing becomes challenging due to large soft-tissue defects, local flap transfer within nontitanium mesh repair areas is the recommended strategy for effectively covering such defects while maintaining the integrity of the titanium mesh.

Takumi and Akimoto [41] described two representative cases of intractable scalp ulcers over the cranial prosthesis that were successfully managed with vascularized calvarial flaps without total removal of the implant. Their experience clearly demonstrated the efficacy of vascularized calvarial flaps in ensuring sufficient blood supply to the graft, reducing the risk of infection, and providing a new tissue bed for the healing of a skin ulcer over a cranial implant for this difficult-to-treat cranial reconstruction. Despite no subsequent re-exposure of the titanium mesh in these patients during follow-up, some uncertainty remains, necessitating comprehensive communication with both patients and their families. In cases characterized by a minimal exposed area and short duration, it is recommended to retain the titanium mesh entirely in the absence of any apparent infection. If there is limited infection or necrotic tissue present beneath the titanium mesh, it is recommended to collaborate with a neurosurgeon in order to remove the affected part of the titanium mesh. Upon opening the titanium mesh, meticulous removal of the necrotic tissue should be performed using a scraper. The endocranium covered by purulent moss should be exposed, and any superficial pus on its surface should be eliminated using a sharp knife, ensuring complete eradication of the pale and brittle granular tissue. If the clipped titanium mesh does not adequately expose or completely eliminate the purulent moss on the surface of the dura mater, more of the titanium mesh can be trimmed. The scope of tissue scraping should be determined by the presence of dense granulation passing through the mesh [42]. The infected purulent moss on the surface of the endocranium often leads to destruction of the granulation passing through the mesh, resulting in observable separation between the affected endocranium and the inner surface of the titanium mesh. After hemostasis is achieved, the wounds are irrigated with hydrogen peroxide solution and normal saline. Subsequently, the wounds are immersed in an iodophor solution at a concentration of 5.5 g/l for 10 to 15 min. The upturned edge of the titanium mesh should be carefully trimmed using specialized titanium mesh scissors, meticulously filed with a bone file, and properly rolled inward to prevent any potential scalp penetration by the sharp components of the titanium mesh after cranioplasty.

If the wound beneath the titanium mesh has completely epithelialized, the incisions can be sutured directly after trimming the edge of the titanium mesh. If there are skin defects after fenestration and debridement, the use of scalp flaps or free flap transplantation can be considered an effective approach for covering the defects. In cases where extensive pus accumulation is observed beneath the titanium mesh, complete removal of the titanium mesh becomes necessary, followed by thorough debridement. Postsurgical treatment should involve proactive measures against infection. When encountering multiple necrotic areas in the flaps with exposed titanium mesh or recurrent exposure, caution must be taken in deciding on the therapeutic strategy aimed at retaining the titanium mesh implant. Due to the thermal and electrical conductivity of titanium mesh, it is recommended to utilize a scalpel during flap elevation whenever possible while employing bipolar electrocoagulation hemostasis to address obvious bleeding points. A low-power electrotome can be used cautiously to gradually separate the flap, ensuring simultaneous cooling with normal saline during this process. It is crucial to avoid any contact between the electrotome or electric coagulation forceps and the titanium mesh to prevent conductive heat generation that may harm subdural brain tissue. When removing the titanium mesh, the utmost priority should be given to protecting the internal meninges and brain tissue from damage, and once damage occurs, prompt utilization of appropriate techniques such as suturing is crucial for effective repair and prevention of postoperative cerebrospinal fluid leakage.

In some cases, the surgical history has involved artificial dural transplantation. If the artificial dural graft exhibits pallor and is accompanied by pus or obvious exudation after the removal of the titanium mesh, it is recommended that the artificial dural graft be replaced with autologous fascia lata to prevent persistent inflammation caused by the infected artificial dural graft and promote wound healing. The retention of the titanium mesh, determination of the fenestration scope, and replacement of the artificial dural graft require the involvement of experienced neurosurgeons to maximize patient outcomes through multidisciplinary cooperation [40].

### Clinical issue 8

Selection of repair techniques for titanium mesh-exposed wounds after cranioplasty.

There are multiple repair techniques available for addressing titanium mesh-exposed wounds, and the selection process should be based on various factors, including the location and size of the exposure, the wound shape, the postoperative duration, the exposure duration, the presence of infection, the local skin conditions, and the patient’s systemic health status. Relevant factors, such as the patient’s willingness to retain the titanium mesh and undergo reoperation for cranioplasty, as well as their risk tolerance, should also inform individualized decision-making.

### Recommendation 8

For patients with skin defects after complete removal of the titanium mesh or fenestration and debridement, if there is no intention for re-cranioplasty or scalp expansion, skin graft transplantation can be considered. (Grade of recommendation: recommended; level of evidence: V.)

### Recommendation 9

It is recommended that for patients who are unable to undergo direct suturing and skin graft transplantation after titanium mesh fenestration or debridement with the retention of the titanium mesh, local flap transfer in the nontitanium mesh area can be considered. (Grade of recommendation: highly recommended; level of evidence: III.)

### Recommendation 10

It is recommended to utilize free flaps with a rich blood supply for the repair of the following conditions. (Grade of recommendation: highly recommended; level of evidence: IV.)

- Thin scalp coverage and retention of all or most of the titanium mesh.
- Large scalp defects and the need for reoperation for cranioplasty after titanium mesh removal.
- Combined with severe infection of the paranasal sinus.

### Recommendation 11

For patients with a short duration of exposure, no or mild infection beneath the titanium mesh, a strong desire to retain the titanium mesh despite the potential risks of skin soft tissue expander infection and titanium mesh retention failure, and an inability to accept local alopecia deformity resulting from skin grafting at the donor area after local head flap transfer, wound irrigation and VSD may be considered to control infection before scalp expansion flap repair. (Grade of recommendation: recommended; level of evidence: III.)

#### Rationale

After complete debridement and retention or removal of the titanium mesh, sometimes the raised scalp flaps can be sutured directly along the original incision. If direct suturing is not achievable, alternative approaches such as flap transplantation or skin grafting can be considered for repair purposes.

**Figure 1:**
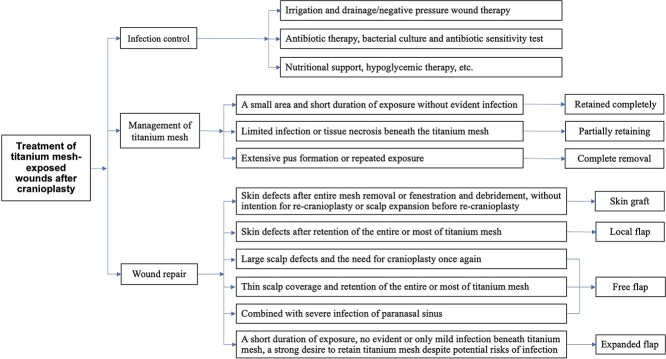
Treatment of titanium mesh-exposed wounds after cranioplasty

- Local skin flap transfer. The design of the flaps should be focused on the nontitanium mesh repair area. For instance, contralateral frontal flaps with supratrochlear vessels or ipsilateral reversed flaps with superficial temporal arteries can be utilized to repair frontal titanium mesh-exposed wounds [43]. Advanced skin flaps with double pedicles can be employed to repair fusiform wounds parallel to the original incisions. Other viable alternatives include S-type or A-T transfer flaps, long arc rotary advanced flaps, or rotation flaps [44]. The flaps should be designed with sufficient size to ensure complete coverage of the wounds and suturing without tension. However, it is noteworthy that when a larger flap is rotated, the donor area often necessitates skin grafts, which may result in alopecia and an unsatisfactory aesthetic outcome.
- Island superficial temporal fascia flaps combined with full-thickness skin grafting. The reparative outcome is satisfactory; however, caution should be exercised when considering the application of this technique in patients who have experienced severance of the temporal muscle during decompressive craniectomy, as it may result in compromised blood supply to the fascial flaps [45].
- Distant pedicle flap transplantation. The application of this technique is primarily limited to cases where there is significant damage to the local flaps and the superficial temporal artery cannot be employed as the recipient vessel due to its impairment. The repair of exposed wounds in the temporal frontal region can be achieved through medial upper arm flaps; however, this approach is generally infrequently employed due to challenges associated with position fixation and substantial patient discomfort.
- Free flap transplantation. Free flaps provide a rich blood supply, effectively protecting the titanium mesh and facilitating the repair of cranial defects [46, 47]. They also create good conditions for subsequent reoperation for cranioplasty [48–50], but the repaired appearance may be bulky and hairless. Furthermore, the successful execution of free skin flap transplantation requires the operator to have high professional skills [51–53]. When utilizing free flaps to repair titanium mesh-exposed wounds, it is common to employ superficial temporal vessels in the recipient area. However, most skull defects after decompressive craniectomy are located mainly in the temporal region, and the incisions form large arcs along the temporal lines. To preserve the remaining blood supply to the residual scalp in the temporal region, flow-through free flap transplantation can be considered. In the presence of frontal sinus, ethmoid sinus, and other paranasal sinus infection, it is necessary to perform complete debridement and meticulous scraping of the sinus cavity, and then employ a tissue flap with rich blood supply (such as a muscle flap) to fill the cavity for effective infection control and optimal wound healing [53]. A clinical study described the use of latissimus dorsi-myocutaneous flaps for the repair of titanium mesh exposure and scalp defects after cranioplasty in 15 patients. All the patients achieved full flap survival, and only one patient had titanium mesh re-exposure and underwent an additional operation to remove the plate [47].
- Expanded flap transplantation. Ji *et al.* [54] reported the use of expanded scalp flaps to repair 13 cases of wounds with exposed titanium mesh after cranioplasty, which can effectively cover the wound and retain the titanium mesh, achieving good function and appearance. The authors considered that this method can be employed to repair titanium mesh-exposed wounds in the following situations: exposure time within 3 months, no apparent signs of infection beneath the titanium mesh, little exudation, normal scalp color and texture surrounding the titanium mesh, tight adhesion between the titanium mesh and endocranium or a small area of separation. In addition, this technique can be used in patients with a strong desire to retain the titanium mesh and a comprehensive understanding of the potential risks such as expander infection, exposure, and failure in retaining the titanium mesh. Liu *et al.* [55] successfully employed the same method to repair titanium mesh-exposed wounds in eight patients. Similarly, Zhao *et al.* [56] effectively treated titanium mesh-exposed wounds with positive microbial culture results by utilizing expanded scalp flaps combined with trypsin irrigation and negative pressure therapy. Based on the abovementioned findings, the application of expanded scalp flaps for repairing titanium mesh-exposed wounds after cranioplasty is a recommended treatment from the professional perspective (Figure 1).

## Conclusions

This consensus was reached on the basis of expert discussions on the mechanism of titanium mesh exposure, preventive measures after cranioplasty, and standard diagnosis and treatment, with the aim of providing a set of preliminary recommendations and proposing corresponding clinical interventions. This consensus serves as a valuable reference for clinicians, particularly in the specialized fields of burn and plastic surgery, as well as wound repair surgery. It holds significant clinical significance in the prevention and management of postoperative titanium mesh exposure while also enhancing postoperative outcomes. Due to the limited number of controlled studies on repairing titanium mesh-exposed wounds, especially the absence of multicenter clinical trials, it is imperative to conduct more high-level evidence-based studies to provide a comprehensive and scientifically sound basis for revising this consensus.

## Consensus consultants

The PLA General Hospital, Xiaobing Fu. The First Affiliated Hospital of Naval Medical University, Zhaofan Xia. Beijing Jishuitan Hospital, Yonghua Sun.

## Team leader

Southern University of Science and Technology Hospital, Yuesheng Huang.

## Consensus committee group

People’s Liberation Army General Hospital of Southern Theater Command, Biao Cheng; Liyuan Hospital of Huazhong University of Science and Technology, Binghui Li; The First Affiliated Hospital of Air Force Military Medical University, Dahai Hu; The Second Affiliated Hospital of Zhejiang University School of Medicine, Guoxian Chen; People’s Hospital of Peking University, Hailin Xu; The First Affiliated Hospital of Jinan University, Hongwei Liu; The First Affiliated Hospital of Army Military Medical University, Guangping Liang, Jiaping Zhang; The First Affiliated Hospital of Nanchang University, Jianhua Zhan; Xiangya Hospital of Central South University, Jingfang Liu, Xiaoyuan Huang, Pihong Zhang; The Second Affiliated Hospital of Air Force Military Medical University, Jing Li; The Tenth Affiliated Hospital of Southern Medical University, Junli Zhou; The Third Hospital in Wuhan City, Shuhua Liu; The Second Affiliated Hospital of Dalian Medical University, Tongbin Chu; People’s Hospital of Xinjiang Autonomous Region, Xiaolong Liu; Qianfo Mountain Hospital of Shandong Province, Yibing Wang; Ruijin Hospital Affiliated to Shanghai Jiao Tong University School of Medicine, Yiwen Liu; The Affiliated Hospital of Nantong University, Yi Zhang; The First Medical Center of General Hospital of PLA, Yufeng Jiang, Rungong Yang; People’s Hospital of Hainan Province, Yunchuan Pan; Southern University of Science and Technology Hospital, Yuesheng Huang; Xiangya School of Public Health, Central South University, Xinyin Wu, Dingkui Sun.

## Methodology expert group

Xiangya School of Public Health, Central South University, Xinyin Wu, Dingkui Sun; West China Hospital of Sichuan University, Cong Wang.

## Writing group

Xiangya Hospital of Central South University, Pihong Zhang, Xiaoyuan Huang; Southern University of Science and Technology Hospital, Yuesheng Huang.

## Clinical problem solicitation expert group

Panelists of the consensus committee group; Chenzhou First People’s Hospital, Xisheng Xu; Xiangya Hospital of Central South University, Pengfei Liang, Jie Zhou; Xiangya School of Public Health, Central South University, Xinyin Wu, Dingkui Sun.

## Evidence evaluation and secretary group

Xiangya Hospital of Central South University, Pihong Zhang, Xiaoyuan Huang, Pengfei Liang, Mitao Huang, Yan Yang, Jie Zhou.

## Registration of consensus and guideline

International Practice Guideline Registry Platform, PREPARE-2022CN647.
